# Effect of granulocyte colony-stimulating factor in experimental methicillin resistant *Staphylococcus aureus *sepsis

**DOI:** 10.1186/1471-2334-4-43

**Published:** 2004-10-18

**Authors:** Emine Alp, Suveyda Gozukucuk, Ozlem Canoz, Beyhan Kirmaci, Mehmet Doganay

**Affiliations:** 1 Department of Infectious Diseases, Faculty of Medicine, Erciyes University, Kayseri, Turkey; 2 Department of Pathology, Faculty of Medicine, Erciyes University, Kayseri, Turkey

## Abstract

**Background:**

Methicillin resistant *Staphylococcus aureus *(MRSA) is the leading pathogenic cause of nosocomial infections, especially in bacteraemia and sepsis. The essential therapy for MRSA infection is glycopeptides. Therapeutic failure can be seen with this therapy and the mortality is still high. The aim of this study was to evaluate the additional effect of G-CSF on the traditional antibiotic treatment in an experimental MRSA sepsis.

**Methods:**

Experimental sepsis was performed in mice by intraperitoneal injection of MRSA isolate. Inoculum dose was estimated as 6 × 10^9^/ml. Mice were randomised for the study into four group; control group (not receive any therapy), G-CSF group (1000 ng/daily, subcutaneously for 3 d), antibiotic group (vancomycin 25 or 50 mg/kg intraperitoneally every 12 hours for 7 d), and vancomycin+G-CSF group (at the same concentrations and duration). Autopsy was done within one hour after mice died. If mice was still alive at the end of seventh day, they were sacrificed, and autopsy was done. In all groups, the effect of G-CSF therapy on the survival, the number of the MRSA colonies in the lung, liver, heart, spleen, and peritoneal cultures, the histopathology of the lung, liver, heart and spleen was investigated.

**Results:**

One hundred and six mice were used. There were no significant differences in survival rates and bacterial eradication in G-CSF group compared with control group, and also in antibiotic +G-CSF group compared with antibiotic alone group. These parameters were all significantly different in antibiotic alone group compared with control group. Histopathologically, inflammation of the lung and liver were significantly reduced in vancomycin (25 mg/kg)+G-CSF and vancomycin (50 mg/kg)+G-CSF subgroups, respectively (p < 0.01). The histopathological inflammation of the other organs was not significantly different in antibiotic+G-CSF group compared with antibiotic group and, also G-CSF group compared with control group.

**Conclusion:**

G-CSF treatment had no additional effect on survival and bacterial eradication in MRSA sepsis in nonneutropenic mice; and only a little effect on histopathology. G-CSF treatment is very expensive, likewise glycopeptides. The more interest in infection control measures, and prevent the spread of MRSA infections is more rational.

## Background

*Staphylococcus aureus *is an extremely virulent pathogen, and causes serious and deep-seated infections (e.g. endocarditis, osteomyelitis) [1]. In recent years, *S. aureus *is the leading pathogenic cause of both community-acquired and nosocomial infections, especially in bacteremia and sepsis [2-4]. Despite advances in antistaphylococcal drugs, *S. aureus *sepsis is one of the most important causes of death [5]. Furthermore, methicillin resistant strains are increasing in community and hospitals during the past decades, and many investigators proposed methicillin resistance as an independent predictor of adverse outcome [6,7]. The essential therapy for methicillin resistant *S. aureus *(MRSA) infections is glycopeptides (vancomycin or teicoplanin). However glycopeptides are intrinsically less effective against staphylococci than are antistaphyloccal β-lactams [1]. This may explain therapeutic failure and high mortality of MRSA infections.

Advances in the pathophysiology of sepsis and septic shock suggest new therapeutic agents and approaches. Granulocyte-colony stimulating factor (G-CSF), a potent stimulator of neutrophil counts and functions, is one of these new strategies in sepsis. Despite a number of studies in sepsis, especially in neonatal sepsis, clinical efficacy of G-CSF is still controversial [8].

The aim of this experimental study was to evaluate the role of G-CSF in the treatment of MRSA sepsis.

## Methods

### Animal care and use

Male BALB-C mice (8–10 weeks old, weighing 20–25 g) were obtained from Erciyes University Hakan Cetinsaya Experimental and Clinical Research Center. The Animal Care Committee of Erciyes University approved the experimental protocol used in this study. The animals were kept in a cage and allowed to feed and drink water.

### Bacterial strain

The clinical MRSA isolate, obtained from a patient's blood and pleural effusion admitted in General Surgical Intensive Care Unit of Erciyes University with nosocomial sepsis, was used. The strain was subcultured on blood agar at 37°C over night. On the day of experiment, bacterial suspension was prepared by sodium chloride 0.9% solution and the concentration was adjusted by spectrophotometer. The bacterial account needed for experimental sepsis was determined by a fore study. The fore study was begun with the inoculum dose of 1 × 10^7^/ml bacterial suspension and gradually increased until experimental sepsis developed. Experimental sepsis was defined as the growth of MRSA in two or more organs. The inoculum dose of this study was estimated as 6 × 10^9^/mL.

### Treatment

First dose of antibiotic was given at the sixth hour of bacterial inoculation. Vancomycin (DBL, 500 mg flacon), diluted in 5% dextrose, was given at 25 mg/kg or 50 mg/kg concentrations intraperitoneally every 12 hours for 7 days. rhG-CSF (Roche, Neupogen flk 30 mu/ 1 mL), diluted in 5% dextrose, was received 1000 ng/daily subcutaneously for 3 days [9].

### Study design

Animals were randomised to four groups; control group, G-CSF group, vancomycin group, and vancomycin +G-CSF group. The last two groups were divided into two subgroups for different dosage of vancomycin (25 mg/kg or 50 mg/kg). Each group had at least 15 mice and were kept in different cage. In control group, only bacteria suspension was given and no treatment was received.

Bacterial suspension was given intraperitoneally to the mice, and when mice died, autopsy was done within one hour in aseptic conditions. If the mice was still alive at the end of seventh day, mice were sacrificed by servical dislocation and autopsy was done. The samples were taken from lung, liver, heart, spleen, and peritoneum for microbiological investigation, and from lung, liver, heart and spleen for histopathological examination. In each group, survival days were noted.

Samples were taken from the organs with swab by one rotation on its axis, and were cultured on blood agar over night. The colonies on the agar were counted. The colonies more than 300 cfu in a plate noted as >300 cfu.

The same pathologist examined tissue samples stained with hematoxylin and eosin. The degree of inflammation was graded by on a scale of 0 to ++++ (0, no inflammation; focal interstitial inflammation +; more diffuse interstitial inflammation ++; intense interstitial inflammation or microabscesses +++; more extensive abscess formation with tissue necrosis ++++) [10]. The pathologist was unaware about the groups.

Survival days, semiquantitative bacterial count and histopathologic findings in the tissues of the treatment groups were compared with the control group.

### Statistics

Survival was assessed using a log-rank test. Fisher's extract test and chi-square test were used to compare the groups. Mann Whitney U test was used to compare the histopathology. A p value less than 0.05 was considered significant.

## Results

**Figure 1:**
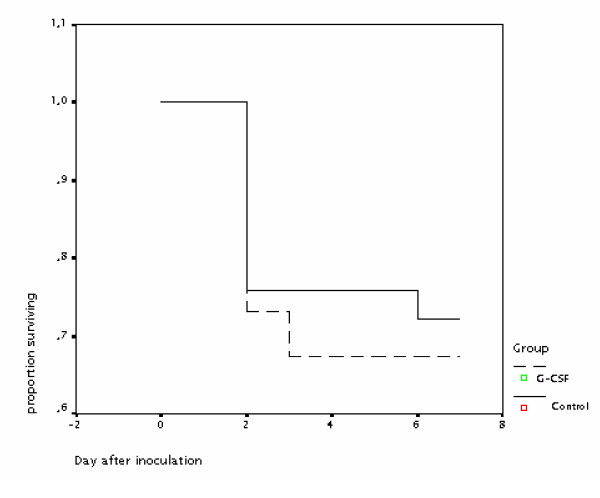
Proportion of animals surviving after inoculation with MRSA in control and G-CSF groups.

**Figure 2:**
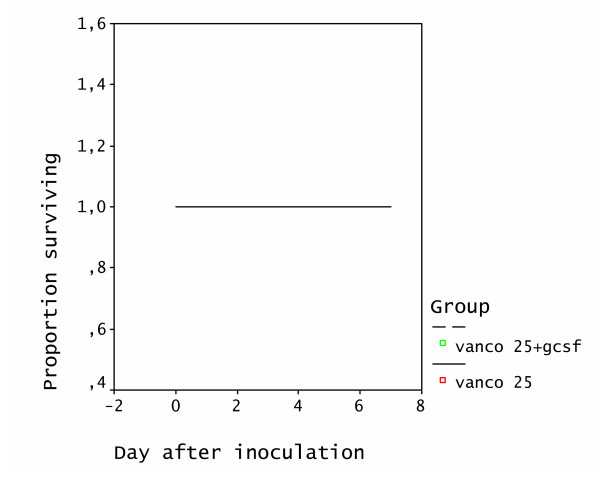
Proportion of animals surviving after inoculation with MRSA in vancomycin 25 mg/kg and vancomycin 25 mg/kg+G-CSF groups

This study included 110 mice. Three mice that developed intraabdominal hemorrhage, and one mouse that developed *Esherichia coli *sepsis were excluded. 106 mice were evaluated. Six mice in control group, six mice in G-CSF group, 15 mice in vancomycin 25 mg, 15 mice in vancomycin 25 mg+G-CSF group, 18 mice in vancomycin 50 mg group and 14 mice in vancomycin 50 mg+G-CSF group were sacrificed at the end of seventh day. The death day and the survival rate of the mice in the groups are shown by the survival curve in figure 1,2,3. The comparison of the groups between G-CSF and control, vancomycin (25 mg/kg) and vancomycin (25 mg/kg)+G-CSF, vancomycin (50 mg/kg) and vancomycin (50 mg/kg)+G-CSF showed no differences (p >0.05).

Culture and histopathology results are shown in Table 1. Antibiotic administration decreased bacterial count, and decreased inflammation rate in the organs. Culture and histopathological results in control and G-CSF group were not statistically different (p > 0.05). However, all these parameters were significantly different in antibiotic groups compared with control group (p < 0.01). Cultures of the organs in antibiotic groups were not statistically different from antibiotic+G-CSF group (p > 0.05). Only the inflammation degree in the lung and the liver were significantly reduced in vancomycin (25 mg/kg)+G-CSF group and vancomycin (50 mg/kg)+G-CSF group, respectively (p < 0.01). The inflammatory changes were not significantly reduced in the other organs in two groups (p > 0.05).

## Discussion

In recent years, MRSA has become widespread around the world, and become highly endemic in some hospitals. In the United States, the proportion of MRSA isolates increased from 2.4% in 1975 to up to 55% in recent years [11,12]. Similarly, in Europe the resistance rates increased from 12.8% to 26.3% [12,13]. Also, it is extremely high (>60%) in some regions of the world [12]. The recent studies, conducted in our hospital, showed 66% methicillin resistance in nosocomial *S. aureus *strains, isolated from the bloodstream infections [2,14]. Unfortunately, community-acquired MRSA also increases during the past decades [15].

G-CSF is a cytokine that stimulates myeloid progenitor cell proliferation and increases the bone marrow storage pool and the number of circulating mature neutrophils, which are important component of the host defense [16]. Also, it enhances neutrophil activities (chemotaxis, phagocytosis, antibody-mediated cytotoxicity, etc.) [17]. An inappropriate endogenous G-CSF response may be associated with an adverse outcome to sepsis. Low serum G-CSF concentrations (0 to 125 pg/mL) on admission are supposed to be associated with fatal outcome in patients with bacterial infections [18]. Investigators proposed to use G-CSF in infections in which neutrophil number and function are important to resolution and survival, also in patients which may have reduced neutrophil numbers or function because of underlying disease or physiologic state. The low toxicity and the beneficial effect on survival in animal studies have led to several clinical trials of rhG-CSF as an adjuvant therapy in treatment of infection in nonneutropenic patients [19,20]. Its beneficial effect was shown in clinical studies in diabetic foot infections, wound infections, extensive burns and fungal infections [21-23]. Also neutrophils in sepsis demonstrate a number of functional abnormalities (e.g. reduced bacterial killing, superoxide production, and migration) [24] and it can be hypothesized that these abnormalities can be corrected with G-CSF.

The previous experimental therapeutic studies were mostly carried out in gram-negative sepsis, and in this experimental study, we investigated the effect of G-CSF in MRSA sepsis. The survival rates were not significantly different in G-CSF group compared with the control group, and also in antibiotic+G-CSF group compared with antibiotic alone group. Likewise, most of the other experimental studies in gram negative sepsis, showed that prophylactic rhG-CSF administration reduced endotoxemia and serum TNF-α levels and also improved cardiac function and survival, whereas therapeutic rhG-CSF (i.e. administered after the onset of infection) did not improve outcome and at very high dosages appeared harmful [25,26]. A recent multicenter, double-blind, randomised and placebo controlled study in patients hospitalised with pneumonia and severe sepsis, demonstrated that G-CSF had no beneficial effect in reducing mortality rates or complications from severe sepsis [27].

In this study, we did not measure the WBC count, so not have subgroups with neutropenia. In an experimental gram-negative sepsis in rabbits, Smith and colleguaes [28] showed the beneficial effect of therapeutic rhG-CSF only in early sepsis due to gram negative bacteria when complicated by leukopenia, no significant difference in nonneutropenic group.

Bacteria are rapidly cleared from blood and tissues following intravenous antibiotic therapy [10]. In our study, antibiotic received group had significantly low bacterial count in the organ cultures compared with the other groups, however G-CSF had no beneficial effect on the bacterial clearance.

The histopathologic findings of invasive *S. aureus *infections are leukocytic infiltration, focal pneumonitis, edema, microabscesses, etc. In an experimental study, significant pathologic changes during and after the elimination of bacteria from the blood and tissues were noted. Also, expression of cytokines (TNF, IL-1, IL-6) was observed in all of the infected tissues, and correlated tissue damage after clearance of bacteria from the blood and tissues[10]. However, in our study, G-CSF significantly reduced only the inflammation in the lung and liver in vancomycin +G-CSF subgroups (p < 0.01). This effect of G-CSF could not be explained. There was no significant effect of G-CSF on the other organs inflammation.

In conclusion, G-CSF treatment had no additional effect on survival and bacterial eradication in MRSA sepsis in nonneutropenic mice; and only a little effect on histopathology. Furthermore, G-CSF treatment is very expensive, likewise glycopeptides. Because of high mortality and morbidity rates and excess costs, more interest in infection control measures, and prevent the spread of MRSA infections is more rational.

## Competing interests

This study was supported by Erciyes University Research Fund. There was no non-financial competing interest.

## Authors' contributions

EA and SG were the primary researchers. OC and BK are the pathologist and examined tissue samples. MD was the director of the study. All authors read and approved the final manuscript.

## Pre-publication history

The pre-publication history for this paper can be accessed here:



## Figures and Tables

**Figure 3:**
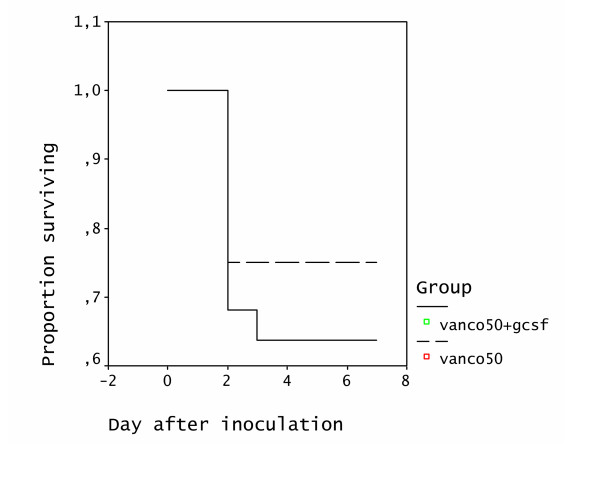
Proportion of animals surviving after inoculation with MRSA in vancomycin 50 mg/kg and vancomycin 50 mg/kg+G-CSF groups

**Table 1:** Cultures and histopathology of study groups

		**Control**	**G-CSF**	**Vanco – 25 mg**	**Vanco 25 mg + G-CSF**	**Vanco – 50 mg**	**Vanco – 50 mg + G-CSF**
		n	n	n	n	n	n

	**Mice**	15	15	15	15	24	22
**Cultures**	**Lung**						
	no growth	-	1	15	15	18	14
	<300 cfu	-	3	-	-	-	-
	>300 cfu	15	11	-	-	6	8
	**Liver**						
	no growth	-	1	15	15	17	14
	<300 cfu	-	2	-	-	1	-
	>300 cfu	15	12	-	-	6	8
	**Heart**						
	no growth	-	1	15	15	18	14
	<300 cfu	-	3	-	-	-	-
	>300 cfu	15	11	-	-	6	8
	**Spleen**						
	no growth	-	-	13	15	13	12
	<300 cfu	-	1	-	-	3	2
	>300 cfu	15	14	2	-	8	8
	**Periton**						
	no growth	-	2	15	15	17	13
	<300 cfu	-	1	-	-	1	1
	>300 cfu	15	12	-	-	6	8
**Histopathology (inflammation degree)**	**Lung**						
	0	0	0	0	0	0	0
	+	2	1	5	1	7	9
	++	7	6	7	6	9	9
	+++	5	7	3	7	8	4
	++++	1	1	0	1	0	0
	**Liver**						
	0	0	0	0	1	1	0
	+	1	4	7	5	12	5
	++	7	2	4	5	9	10
	+++	4	6	4	4	2	7
	++++	3	3	0	0	0	0
	**Heart**						
	0	1	0	8	8	10	10
	+	9	11	7	6	12	7
	++	5	2	0	1	0	5
	+++	0	0	0	0	0	0
	++++	0	0	0	0	0	0
	**Spleen**						
	0	0	1	0	0	1	1
	+	0	2	7	5	13	8
	++	5	4	7	6	7	8
	+++	4	6	1	4	2	5
	++++	6	2	0	0	1	0

**Inflammation degree **No inflammation 0

Focal interstitial inflammation +

More diffüse interstitial inflammation ++

Intense interstitial inflammation veya microabscesses +++

More extensive abscess formation with tissue necrosis ++++
